# Determinants of sexual health knowledge in adolescent girls in schools of Riyadh-Saudi Arabia: a cross sectional study

**DOI:** 10.1186/1472-6874-13-19

**Published:** 2013-04-15

**Authors:** AlJohara M AlQuaiz, Ambreen Kazi, Maha Al Muneef

**Affiliations:** 1 Princess Nora Bint Abdullah Women Health Research Chair, College of Medicine, King Saud University, PO Box 231831, Riyadh 11321, Saudi Arabia; 2 National Family Safety Program & Infectious Diseases, King Saud Bin Abdul Aziz University for Health Sciences, Riyadh, Saudi Arabia

**Keywords:** Sexual health knowledge, Female, Adolescents, Parent’s role, Friends, School curriculum

## Abstract

**Background:**

There are many social and cultural factors affecting the sexual knowledge of adolescents. This study measured the sexual health knowledge level of adolescents and identified its association with role of parents, friends and school environment in adolescent girls in Riyadh, Saudi Arabia.

**Methods:**

Four hundred and nineteen Saudi female students belonging to intermediate and secondary grades were randomly selected from four public and private girl schools. 255 (69.8%) students were ≤15 years and 164 (39.2%) were >15 years. A self-administered structured questionnaire comprising of socio-demographic information, role of parents and teachers, availability of school curriculum on sexual health was used. Sexual health knowledge was assessed through questions on identification of physical changes during puberty for ≤15 years and separate questions on sexually transmitted infections for >15 years.

**Results:**

54% of ≤15years and 70.7% of >15 years had poor sexual health knowledge. Multivariate analysis found determinants for poor sexual health education in ≤15years are: lower education level of both parents (OR 10.87; 95% CI 2.44–48.38), second birth order or more (OR 2.32; 95% CI 1.24–4.33) and absence of school curriculum on sexual health (OR 0.56; 95% CI 0.33–0.95). Determinants for >15 years of age are : mothers with low literacy (OR 3.08, 95% CI 1.42–6.71), as for sources of poor sexual knowledge : parents (OR 10.10; 95% CI 2.70–37.74), schools (OR 6.95; 95% CI 1.95–24.78) maids (OR 4.57; 95% CI 1.26–16.59) and media (OR 5.12; 95% CI 1.29–20.07) were statistically significant factors.

**Conclusion:**

Government agencies with collaboration of all stake holders should develop policies and programs for implementing and evaluating integrated and comprehensive sexual educational programs for adolescents in Saudi Arabia.

## Background

Knowledge on sexual health encompasses information related to sexuality, reproductive and sexual health care problems and services available, autonomy over choice of partner and decision regarding family planning [1]. Correct knowledge regarding sexual health is important for all especially adolescents, as they are vulnerable to adopt negative behaviors if not guided properly and at the right age [2]. Various similar and dissimilar factors contribute towards sexual health knowledge and behavior of adolescents across the globe [3]. These may range from gender differences [3], parental supervision [4,5], role of community [4,5] peer influence [5], school environment [4,5], economic concerns [3], and neighborhood surroundings [6] to ethnicity [7].

Improper information on sexual health may lead to various types of health risks and social problems. Health risks may include; acquiring sexually transmitted diseases including HIV infection [8], early pregnancies [9], unsafe abortions [9], adverse birth outcomes due to consanguineous marriages [10], maternal morbidity and mortality [8. 9]. Social risks may involve initiation of smoking [11], drug abuse [12], physical and sexual violence [13] young age marriages [4], and suicide [14]. Adolescent’s low level of education predisposes them to adopt negative behaviors like smoking and drug abuse, which further aggravate their risky sexual behavior [3].

Males and females have different level of exposure to various sources of information which affect their sexual health knowledge. Females belonging especially to Middle East region are at more risk as they have less access to information sources outside their homes, whatever information they receive is through their mothers, siblings or friends [15].

Majority of studies conducted world over are based on sexual health behavior of adolescents rather than knowledge [3]. However, in countries like Saudi Arabia before exploring the behaviors it is essential to identify the important factors affecting the sexual health knowledge. The findings of such studies will help in designing successful programs for adolescent’s health specially girls [16]. It has been sometime since Saudi Arabia (SA) has informally introduced sexual health topics within the school curriculum. A study was conducted to assess sexual health knowledge, attitudes and resources among adolescent girls belonging to public and private schools in Riyadh, SA. This study identified 42% of students discuss sexual topics with their friends, 15.8% with their parents and 17% with their household servants [17]. This study however was not able to identify which are the important determinants for sexual health knowledge of adolescent girl students. We hypothesize that factors related to parents, friends and school will be significant in determining the sexual knowledge of adolescents in Riyadh, SA.

## Methods

In Saudi Arabia, both public and private schools are functional and education begins at the age of six years. It includes three educational levels; primary school level (grade 1 to 6) attended by 6 to 12 years old children, intermediate school level (grade7 to 9) attended by 13 to 15 years old adolescents and secondary school level (grade 10 to 12) attended by 16–18 years old adolescents. The students of intermediate and secondary school level were included in this study.

### Sample size and sampling methodology

For risk factors of sexual health knowledge, assuming a type- I error of 0.05, type-II error of 0.20 (power of 0.80) and 20% difference of prevalence of risk factors in those with appropriate and inappropriate knowledge level we needed 400 girl students to participate in the study.

A complete list of schools was received from the Education Ministry and four girls schools, belonging to both, public and private sector were randomly selected from four administrative regions of Riyadh city. Selection of schools was also based on co-operation from the administration and teachers and feasibility to conduct a research study. Only female schools could be targeted as permission for male schools was not granted. An approval was taken from the Internal Review Board of the University as well as from Riyadh Regional Education Supervision Office (Education Ministry), school authorities and the students (school administration gave the consent on behalf of parents). Schools and students were assured of keeping this information confidential and utilizing it for research purpose only.

### Procedure

It was a cross sectional study and students were invited to answer a self-administered questionnaire. There are around 5 to 6 classes in each level (intermediate and secondary). All classes belonging to same level were coded and mentioned on separate slips of paper. Two classes per level were randomly selected (through slip method) and all students (average 25 per class) in the selected class were invited to participate in the study. There were no refusals. Students were given half an hour to answer all questions and teachers consented to supervise the process. No student was allowed to discuss or take the questionnaire outside the class. All questions were handed back to the teachers in sealed envelopes and were opened at data editing and entry time.

### Measures

The questionnaire comprised of information on independent variables related to socio-demographics, different information sources on sexual health knowledge, school curriculum, role of teachers and role of parents. Questions related to parents included their educational level and their role in discussing sexual health knowledge topics. Section related to school included questions on type of school (public or private), availability and content of the curriculum and role of teachers in discussing sexual health topics. Question related to different sources of information on sexual health included parents, school, friends, maids and media sources. Student’s satisfaction and comfort level with different sources of information was also assessed.

As the classes targeted were intermediate and secondary grades which have different age groups, therefore separate question was asked to measure the dependent (outcome) variable on knowledge of sexual health. After careful discussion with experts (teachers and family physicians) and keeping the cultural context in view, specific criteria to define “appropriate sexual knowledge level” was established.

Operational definition for appropriate level of sexual knowledge of ≤ 15 years’ adolescent girls was taken as “who marks correctly more than 50% physiological changes occurring during puberty separately in boys and girls”. The physiological changes mentioned in the questionnaire among boys were: *change in voice, appearance of hairs on the face, arm pits and genitalia, apparent change in body physique.* Physiological changes among girls were: *appearance of hair in arm pits and genitalia, start of menstrual cycle, growth of breasts in size.* Sum of these two variables was converted to a binary variable with 0 having appropriate knowledge and 1 having poor knowledge on sexual health.

Operational definition for appropriate level of sexual knowledge of > 15 year adolescent girls was taken as “who marks correctly all the four common sexually transmitted infections”. Sexually transmitted infections comprised of: Acquired Immune deficiency syndrome (AIDS), Syphilis, Gonorrhea, Hepatitis B. Finally a binary variable was made with 0 having appropriate knowledge and 1 having poor knowledge on sexual health.

### Data analysis

Data was analyzed using SPSS computer statistical software package (IBM SPSS statistics version 19). Data was divided into two groups based on age; ≤15 years and >15 years. The dependent variable on sexual knowledge was divided into poor and appropriate sexual health knowledge levels. Proportions were calculated for both dependent and independent variables. The level of statistical significance was considered as p < 0.05. Univariate analysis was done to identify significant biological plausible variables. Variables were further checked for multi-colinearity. Two separate multivariate logistic regression model were developed to identify important factors for poor sexual health knowledge among ≤15 years and > 15 years, after adjusting for socio demographic, parental and school related variables.

## Results

### Age group ≤15 years

A total of 255 students belonged to ≤15 years age group. Descriptive analysis found 54.1% (138) with *poor* and 45.9% (117) with *appropriate* sexual health knowledge. Majority of students had fathers with graduate and above level of education (73.2% with poor and 81.2% with appropriate knowledge level). Similarly, majority students had sister as sibling (87.6% with poor and 94.9% with appropriate knowledge level). Almost all students were living with both of their parents together (95.6% with poor and 89% with appropriate knowledge level). Almost equal numbers of students were from public and private sector schools (Table 1).

**Table 1:** Univariate analysis showing association of socio-demographic, parenting and schooling with poor sexual health knowledge

	**Age group ≤15 years**			**Age group >15 years**		
	**(n = 255)**			**(n = 164)**		
**Variables**	**Poor sexual health knowledge n = 138 (54.1%)**	**Appropriate sexual health knowledge n = 117 (45.9%)**	**Unadjusted OR (95% CI)**	**Poor sexual health knowledge n = 116 (70.7%)**	**Appropriate sexual health knowledge n = 48 (29.3%)**	**Unadjusted OR (95% CI)**
**Father’s Education level**						
Graduate/postgraduate	101 (73.2)	95 (81.2)	1 (reference)	85 (73.3)	37 (77.1)	1 (reference)
Secondary/primary	37 (26.8)	22 (18.8)	1.58(0.87–2.87)	31 (26.7)	11 (22.9)	1.23(0.57–2.70)
**Mother’s Education level**						
Graduate/post graduate	76 (55.1)	74 (63.2)	1 (reference)	48 (41.4)	29 (60.4)	1 (reference)
Secondary/primary	62 (44.9)	43 (36.8)	1.40(0.48–2.32)	68 (58.6)	19 (39.6)	2.16(1.08–4.30)
**Having sister as sibling**						
One and more	120 (87.6)	111 (94.9)	1 (reference)	108 (93.1)	47 (97.9)	1 (reference)
None	17 (12.4)	6 (5.1)	2.62(0.99–6.88)	8 (6.9)	1 (2.1)	3.48(0.42–28.6)
**Birth Order**						
Eldest among siblings	96(69.6)	98 (83.8)	1 (reference)	89 (76.7)	33 (68.8)	1 (reference)
2nd or later in birth order	42 (30.4)	19 (16.2)	2.26(1.22–4.17)	27 (23.3)	15 (31.3)	0.67(0.32–1.41)
**Living with parents**						
Both	129 (95.6)	104 (88.9)	1 (reference)	104 (89.7)	44 (91.7)	1 (reference)
Single	6 (4.4)	13 (11.1)	0.37(0.14–1.01)	12 (10.3)	4 (8.3)	1.27(0.38–4.2)
**Source of sexual health knowledge**						
Friends	8 (5.9)	8 (6.8)	1 (reference)	6 (5.2)	12 (25)	1 (reference)
Parents	51 (37.80	40 (34.2)	1.27(0.44–3.69)	37 (32.2)	7 (14.6)	10.57(3.0–37.6)
School	24 (17.8)	18 (15.4)	1.33(0.42–4.23)	31 (27)	11 (22.9)	5.63 (1.7–18.6)
Maid	38 (28.1)	30 (25.6)	1.26(0.42–3.77)	23 (20)	10 (20.8)	4.6(1.34–15.73)
Media sources^1^	14 (10.4)	21 (17.9)	0.66(0.20–2.19)	18 (15.7)	8 (16.7)	4.5(1.24–16.28)
**Comfortable discussing sexual matters with:**						
Close friend of same age	45 (34.4)	44 (38.6)	1 (reference)	64 (56.6)	22 (47.8)	1 (reference)
Parents	20 (15.3)	21 (18.4)	0.93(0.44–1.95)	15 (13.3)	10 (21.7)	0.51(0.20–1.31)
Relatives/siblings	31 (23.7)	27 (23.7)	1.12(0.58–2.18)	25 (22.1)	8 (17.4)	1.07(0.42–2.72)
Maids	35 (26.7)	22 (19.3)	1.56(0.79–3.05)	9 (8)	6 (13)	0.52(0.16–1.61)
**Parents role in sexual knowledge**						
Major	116 (84.1)	106 (90.6)	1 (reference)	97 (83.6)	31 (64.6)	1 (reference)
Minor	22 (15.9)	11 (9.4)	1.83(0.85–3.95)	19 (16.4)	17 (35.4)	0.35(0.16–0.77)
**Attending**						
Public School	76 (55.1)	68 (58.1)	1 (reference)	77 (66.4)	32 (66.7)	1 (reference)
Private school	62 (44.9)	49 (41.9)	1.13(0.69–1.86)	39 (33.6)	16 (33.3)	1.01(0.49–2.07)
**School curriculum discusses sexual topics**						
Yes	68 (49.3)	40 (34.2)	1 (reference)	61 (52.6)	22 (45.8)	1 (reference)
No	70 (50.7)	77 (65.8)	0.53(0.32–0.88)	55 (47.4)	26 (54.2)	0.76(0.39–1.49)
**School curriculum adequate**^**2**^						
Yes	45 (66.2)	31 (77.5)	1 (reference)	52 (85.2)	20 (90.9)	1 (reference)
No	23 (33.8)	9 (22.5)	1.76(0.72–4.31)	9 (14.8)	2 (9.1)	1.73(0.34–8.72)
**Role of Teachers**						
Co-operative	85 (61.6)	71 (60.7)	1 (reference)	69 (59.5)	28 (58.3)	1 (reference)
Un co-operative	53 (38.4)	46 (39.3)	0.96(0.58–1.59)	47 (40.5)	20 (41.7)	0.95(0.48–1.88)

^1^Books, magazines, internet.

^2^Only those included who said curriculum discusses sexual topics.

Univariate analysis found that adolescents not having any sister as sibling (OR 2.62; 95% CI 0.99–6.88) are at more risk of poor sexual health knowledge as compared to those who had sisters. Similarly adolescents second or more in birth order were at more risk of poor sexual knowledge level (OR 2.26; 95% CI 1.22–4.17) as compared to eldest child in the family. Students who had fathers with low level of education were at risk for poor sexual health knowledge, although results were statistically insignificant (OR 1.58; 95% CI 0.87–2.87). Students who perceived their school curriculum insufficient to discuss sexual health topics were at less risk for poor sexual health knowledge (OR 0.53; 95% CI 0.32–0.88) (Table 1).

Parents education, birth order and school curriculum were entered in the multivariate model 1 (Table 2). Fathers education was marginally significant and associated with poor level of sexual health knowledge (OR 0.32; 95% CI 0.09–1.08) whereas mothers education was not associated with sexual knowledge (OR 0.83; 95% CI 0.45–1.55). However when father and mother’s education were entered together as an interacting variable in the multivariate model, the interaction term in the model found having both father and mother with low education level at much higher risk of poor sexual health knowledge (OR 10.87; 95% CI 2.44–48.38).

**Table 2:** Multivariate logistic regression showing adjusted odds ratio between socio-demographic and curriculum and poor sexual health knowledge in ≤15 year adolescents

**Variables**	**Adjusted odds ratio**	**95% CI**	**p value**
**Father’s Education level**			
Graduate/postgraduate	1 (reference)		
Secondary/primary	0.32	0.09–1.08	0.06
**Mother’s Education level**			
Graduate/post graduate	1 (reference)		
Secondary/primary	0.83	0.45–1.55	0.56
**School Curriculum discusses sexual topics**			
Yes	1 (reference)		
No	0.58	0.33–0.95	0.04
**Birth Order**			
Eldest among siblings	1 (reference)		
Being2nd or more in birth order	2.25	1.19–4.26	0.01
**Interaction term between**			
Low fathers education with Low mothers education	10.87	2.44–48.38	<0.01

Birth order of two or more in number (OR 2.32; 95% CI 1.24–4.33) was a risk factor for poor knowledge, whereas students who thought school curriculum do not discuss sexual health topics were at less risk for poor sexual health knowledge (OR 0.56; 95% CI 0.33–0.95) (Table 2).

### Age group >15 years

A total of 164 students were >15 years of age. 70.7% (116) of students had *poor* and 29.3% had *appropriate* sexual health knowledge. More than 70% of students had fathers with post graduate and graduate level of education. 90% of students had one or more number of sisters as sibling. Majority of students belonged to second or more order of birth (76.7% with poor and 68.8% with appropriate knowledge level) (Table 1).

Univariate analysis found that students who have mothers with low level of education are at more risk (OR 2.16; 95% CI 1.08–4.30) as compared to those who had mothers with graduate or post-graduate level of education. Sources of information found that who refer to parents (OR 10.57; 95% CI 3.0–37.6), school (OR 5.63; 95% CI 1.7–18.6), maids (OR 4.6; 95% CI 1.34–15.73) and media sources (OR 4.5; 95% CI 1.24–16.28) are all at risk of poor knowledge level. Students who thought parents have minor role in discussing sexual information were at less risk (OR 0.35; 95% CI 0.16–0.77) as compared to those who thought parents have major role (Table 1).

Multivariate Model 2 (Table 3) for >15 years found mothers low education level, sources of information and role of parents as important determinants for poor sexual knowledge. Students having mothers with secondary or low level of education (OR3.08, 95% CI 1.42–6.71 and having the following as source of information; parents (OR 10.10; 95% CI 2.70–37.74), school (OR 6.95; 95% CI 1.95–24.78), maids (OR 4.57; 95% CI 1.26–16.59) and media (OR 5.12; 95% CI 1.29–20.07) were at more risk of poor sexual health knowledge. Parents having a minor role in discussing sexual knowledge was a protective factor (OR 0.35, 95% CI 0.15–0.85) as compared to parents major role.

**Table 3:** Multivariate logistic regression showing adjusted odds ratio between socio-demographic, information sources and poor sexual health knowledge

**Variables**	**Adjusted odds ratio**	**95% CI**	**p value**
**Mother’s Education**			
Graduate & post graduate	1 (reference)		
Secondary & primary level	2.44	1.14–5.21	0.02
**Source of Information on sexual knowledge**			
Friends	1 (reference)		
Parents	10.10	2.70–37.74	<0.01
School	6.95	1.95–24.78	<0.01
Maid	4.57	1.26–16.59	0.02
Media sources	5.12	1.29–20.07	0.02
**Role of Parents in sexual knowledge**			
Major	1 (reference)		
Minor	0.36	0.15–0.85	0.02

## Discussion

Our study identified that despite keeping very simple assessment criterion for measuring sexual health knowledge; more than 50% of adolescents in both less and above 15 years had poor sexual health knowledge. Similar results have been reported not only from other provinces [18] in Saudi Arabia but generally from the whole Middle East region [19]. This reflects that for a long time no concrete measures have been taken to impart important and basic information on sexual health to adolescents or to other age groups [15].

Important determinants of sexual knowledge were identified through two separate models for ≤15 years and >15 years of age. The major factors for ≤15 years were; *parents education, demographics and role of school*, whereas for >15 years; *mothers education, role of parents and different sources of information for sexual knowledge* were important factors.

Results of our study support the findings of other research papers which have identified parent’s education as an important factor for adolescent’s sexual knowledge and behavior [20]. In our case where both parents are uneducated, interaction term found almost eleven times more risk of poor sexual health knowledge. Educated parents tend to follow constructive communicating style which helps in developing trustworthy relationship with their adolescents [21]. Parents if perform their role positively are not only the guardians, but they are the role models, guide, teacher and a friend for their children [22]. Both poor communication by parents [23-25] and poor parental practices [26] are established risk factors for negative sexual behavior among children. In addition, parents with low level of education do not prove to be a good source of information on sexual knowledge [27]. Research study in Saudi Arabia found adolescent girls follow unhygienic style during their menstrual cycle and though education level was not measured but it is assumed that their mothers, sisters or friends (as source of information) must be uneducated or unaware of the adverse health consequences [28]. Hence, for the success of any intervention targeted towards building the sexual health knowledge of adolescents it is important to educate, train and involve the parents from the beginning [29,30]. However, for >15 years, mothers educational level alone (and not fathers) was identified as important factor may be as older girls are more close and open with their mothers in discussing topics like sexual health [27,31]. Many programs focusing on education are currently working in the Middle East countries [15], these programs work closely with parents as no change is possible until parents and family is engaged. Similar programs can help in achieving better results in Saudi Arabia as well.

For older adolescents, parent’s role was not found as an important determinant of sexual knowledge. This can be explained by the fact that as adolescents grow in age and with more exposure to outside world, parents alone not remain the major source of information or discussion, they access information and have discussion with sources like friends outside their home [32]. Research studies conducted in Egypt, Morocco and Turkey have identified friends as the most important source for adolescents in building their knowledge on sexual health [15], although in few cases friends were labeled as unreliable source of information. Therefore it is important for parents to know who and where the friends are as peer influence is simultaneously identified as a risk factor for negative health behavior [4].

School has always been considered as an important source for building knowledge on sexual health [33]. Survey on school children in Lebanon has identified the role of schools and community to be even stronger than parent’s role [34]. However, our study found school curriculum as a risk factor for poor sexual health knowledge. This may be because curriculum is incomplete or irrelevant, or teachers and staff are not trained properly to impart information or students are not comfortable in discussing the subject with the teachers [34,35]. In addition, teachers at times are reluctant and not open to the subject. The type of school was also not significantly associated with the knowledge level suggesting that both public and private schools need to revise their strategy for imparting sexual knowledge to students. Studies from Morocco and Egypt have identified girl students not attending schools are at higher risk of early marriage and pregnancy (as early as 15) therefore apart from the school curriculum and teachers role, attending school alone can help in improving the health of adolescent girls. On other hand, studies are available which have found no difference in the sexual behavior of students taught by trained or untrained teachers [36]. So maybe it is the cumulative change which is required to improve the knowledge of students on sexual health.

Two other sources of information, maids and internet (including magazines and books) though common but were found as a risk factor for poor knowledge on sexual health. Maids in Arab culture are from diverse background and majority of them are uneducated, hence information received through them may not be counted as reliable. Recently, internet and media sources have become a convenient way of accessing information on any topic, however they lack the explanation which is required to understand them and at times prove to be harmful rather than beneficial for young adolescents [32,37]. Morocco, Lebanon and Egypt and some other Arab countries have taken an initiative in launching educational websites providing free, safe and correct information on sexual knowledge. It should be the responsibility of health and education Ministry to launch local websites providing correct information on this topic. It will save the youth from getting misguided and help disseminating the information to parents and other adult family members as well. In addition, parents not need to be a good source of information alone, they should be vigilant about other resources of information as well [4].

Children belonging to large families are not only exposed to health risks but have less chance of communicating and interacting with their parents. Our study supports the finding on eldest child being the first one tends to get more attention and hence more comfortable in discussing and gaining knowledge on topics like sexual health. Appropriate number and spacing between the children can help in developing strong relationship between the parents and every child [38].

The study has limitations in terms of measurement criteria set for poor and appropriate knowledge level. Reliable measurement tool like 24 item Miller-Fisk sexual knowledge questionnaire [39] is used to measure knowledge, however it includes questions focusing on topics like menstruation, pregnancy and fertility, majority of them are above the knowledge level of adolescent girls in Saudi Arabia as they have no prior formal science/medical education. We have set the criteria for appropriate knowledge based on basic information and cultural context which is different than the International validated questionnaire. Hence study may not be generalizable to other societies where more detailed information on sexual knowledge is given from childhood and studies are conducted on sexual behaviors rather than knowledge [2]. Another limitation of our study is that we have not been able to study the effect of poverty, neighborhood, religion and ethnicity on sexual knowledge [7]. In certain cultures, discussing sexual health is considered as a religious offence, hence there is no forum available to disseminate or achieve correct and complete knowledge regarding sexual health [30]. Conducting a research study on sexual knowledge level was a challenge in a society like Saudi Arabia and we had to restrict the questionnaire accordingly. The only appropriate way of approaching the adolescents was through schools, therefore those who do not attend school or were absent on that specific day were not included in the study. In, addition since this was a self-administered questionnaire hence students may have been under or over-reporting the information. This may have affected the results to which we are unaware.

## Conclusion

Education level, parents, friends and school are important determinants affecting the sexual knowledge level of adolescents. Research studies have identified that age for puberty for girls is decreasing worldwide exposing the adolescents to various risk factors [40]. Hence in countries like SA, delivery of topic specific, cultural oriented and relevant sexual knowledge at family and school level should be initiated from younger age.

We recommend that Government agencies with the help of all stake holders [adolescents, parents, health care providers, educators, Government agencies] should develop policies and programs for implementing and evaluating integrated and comprehensive sexual educational programs throughout the educational institutes in the Kingdom of Saudi Arabia. Committees at school level comprising of parents, health care providers, religious leaders and teachers should design a culturally acceptable curriculum and adopt the practical ways of disseminating it. Similarly, religious leaders through special classes for girls and boys and in presence of parents can discuss the core important and sensitive topics which otherwise build mis-concepts about sexual health. They can help in clarifying any myths associated with sexual health in the light of religion. All these steps will help not only in reducing the health risks associated with misinformation but will help in building a healthy nation.

## Competing interests

All authors declare that they have no competing of interest.

## Authors’ contribution

Dr JMA has special interest in Women’s physical, social and psychological health. She contributed to the initial concept and design of the study. She co-ordinated and supervised the field work, data editing, entry, and write up process. Dr AK has special interest in the social and mental health of women and contributed to the analysis and the write up of the paper. Dr MAM contributed in the proposal development and manuscript write up. All authors have read and approved the final manuscript.

## Pre-publication history

The pre-publication history for this paper can be accessed here:

http://www.biomedcentral.com/1472-6874/13/19/prepub
